# A Global Perspective on Sulfur Oxide Controls in Coal-Fired Power Plants and Cardiovascular Disease

**DOI:** 10.1038/s41598-018-20404-2

**Published:** 2018-02-08

**Authors:** Cheng-Kuan Lin, Ro-Ting Lin, Pi-Cheng Chen, Pu Wang, Nathalie De Marcellis-Warin, Corwin Zigler, David C. Christiani

**Affiliations:** 1 Department of Environmental Health, Harvard T.H. Chan School of Public Health, 665 Huntington Avenue, Building 1, Room 1406, Boston, Massachusetts, 02115 USA; 2 Department of Occupational Safety and Health, China Medical University, 91 Hsueh-Shih Road, Taichung, 40402 Taiwan; 3 Department of Environmental Engineering, Cheng Kung University, 1 University Road, Tainan City, 701 Taiwan; 4 Institute of Science and Development, Chinese Academy of Sciences, No.15 Zhong Guan Cun Bei Yi Tiao Alley, Haidian District, Beijing, 100190 China; 5 Department of Mathematics and Industrial Engineering, Polytechnique Montréal, 2900, boul. Édouard-Montpetit, Montréal, Québec H3T 1J4 Canada; 6 Department of Biostatistics, Harvard T.H. Chan School of Public Health, 655 Huntington Avenue, Building 2, 4th Floor, Boston, MA 02115 USA; 7 Department of Epidemiology, Harvard T.H. Chan School of Public Health, 665 Huntington Avenue, Building 1, Room 1401, Boston, Massachusetts, 02115 USA

## Abstract

Sulfur oxides (SO_x_), particularly SO_2_ emitted by coal-fired power plants, produce long-term risks for cardiovascular disease (CVD). We estimated the relative risks of CVD and ischemic heart disease (IHD) attributable to SO_x_ emission globally. National SO_x_ reduction achieved by emissions control systems was defined as the average SO_x_ reduction percentage weighted by generating capacities of individual plants in a country. We analyzed the relative risk of CVD incidence associated with national SO_x_ reduction for 13,581 coal-fired power-generating units in 79 countries. A 10% decrease in SO_x_ emission was associated with 0.28% (males; 95%CI = −0.39%~0.95%) and 1.69% (females; 95%CI = 0.99%~2.38%) lower CVD risk. The effects on IHD were > 2 times stronger among males than females (2.78%, 95%CI = 1.99%~3.57% vs. 1.18%, 95%CI = 0.19%~2.17%). Further, 1.43% (males) and 8.00% (females) of CVD cases were attributable to suboptimal SOx reduction. Thus, enhancing regulations on SO_x_ emission control represents a target for national and international intervention to prevent CVD.

## Introduction

CVD has been a leading cause of death globally for decades^1^. Treating CVD is costly, especially in the United States (US). For the US, the burden of medical cost for CVD was 656 billion United States Dollars (USD) in 2015 and is projected to reach 1,208 billion USD in 2030^2^.

Controlling emissions from power-generating plants is important for human health as well as climate. Among the health problems linked to sulfur oxides (SO_x_) exposures in air is cardiovascular disease (CVD)^3,4^. Various air pollutants initiate and promote atherosclerotic progression^5,6^ and are associated with transient increases in plasma viscosity and thrombus formation^7^. Clear links have been drawn between SO_2_ and CVD^8,9^. Indeed, total suspended particles (TSP) and SO_2_ are associated with changes in vasomotor tone^10^ and thus alter heart rate^11,12^ and cardiac function^13^. Such mechanisms may underlie the association between SO_2_ and CVD.

Coal-fired power-generating facilities have long been known to emit pollutants that fuel climate change and adversely impact human health. Among these emissions are SO_x_, including sulfur dioxide (SO_2_). Global SO_2_ emissions, measured by the bottom-up mass balance method, peaked in the early 1970s and decreased for decades^14^. After the 2000s, these emissions increased again, mostly from developing countries^15^. The majority of SO_x_ in the air is anthropogenic emission from coal-fired power plants^16^. For example, in the US, 65% of SO_2_ emission were from electric utilities, and more than 90% of those were coal-fired power plants^17^. Similarly, in the European Union, more than 70% of the emission was from electricity sectors^18^.

To control these emissions, dozens of methods with relatively high efficiencies have been developed for fitting of coal-fired power plants. SO_x_ emissions are determined by (1) the sulfur content in coals burned and (2) the emission control system used^19^. The principle technology of emission control systems is the use of sorbents to scrub SO_x_ from the flue gas, called a flue gas desulfurization (FGD) system. Another method to reduce SO_x_ emission is the use of low sulfur coal, such as sub-bituminous coal mined in the Powder River Basin of Montana and Wyoming^19^. However, this method is not efficient and has a limited application globally. Indeed, FGD products represent an efficient and economically feasible approach to control emissions on a large scale. The cost to retrofit US plants with FGD equipment was estimated at about $407 (2008 USD) per kilowatt (kW) for a 500-megawatt (MW) plant in 2009; this cost escalates yearly by $16^20^. For most nations, coal-fired power plants are either state-owned or government-funded, giving governments direct authority on implementing emission controls; privately-owned power-generating units can be required to follow emissions regulations.

Here, we estimated the relative risks and incident cases of CVD, particularly ischemic heart disease (IHD), attributable to SO_x_ emission from coal-fired power plants from a global perspective. This study sought to determine the potential reduction in preventable CVD that could be attributed to reduced global SO_x_ emissions.

## Results

Data on the coal capacities of power plants across the globe were derived from the Utility Data Institute World Electric Power Plants Data Base (UDI WEPP)^21^. We identified a total of 13,581 generating units in 79 countries that used coal as the primary energy source (Table 1). Most were in Europe (*N* = 36), the Americas (*N* = 12), and the Western Pacific (*N* = 11).

**Table 1:** Countries included in the analysis, by geographical region (N = 79).

Regions	N	Countries
Africa	10	Botswana, Madagascar, Mauritius, Namibia, Niger, Nigeria, Senegal, South Africa, Swaziland, Tanzania, Zambia, Zimbabwe
Americas	12	Argentina, Brazil, Canada, Chile, Colombia, Dominican Republic, Guatemala, Honduras, Mexico, Panama, Peru, United States
Europe	36	Albania, Austria, Belgium, Bosnia and Herzegovina, Bulgaria, Croatia, Czech Republic, Denmark, Finland, France, Germany, Greece, Hungary, Ireland, Israel, Italy, Kazakhstan, Kyrgyzstan, Macedonia, Moldova, Montenegro, Netherlands, Norway, Poland, Portugal, Romania, Russia, Serbia, Slovakia, Slovenia, Spain, Sweden, Turkey, Ukraine, United Kingdom, Uzbekistan
South East Asia	7	Bangladesh, India, Indonesia, Myanmar, North Korea, Sri Lanka, Thailand
Western Pacific	11	Australia, Cambodia, China, Japan, Malaysia, Mongolia, New Zealand, Philippines, South Korea, Taiwan, Vietnam
Eastern Mediterranean	3	Morocco, Pakistan, Syria

**Figure 1:**
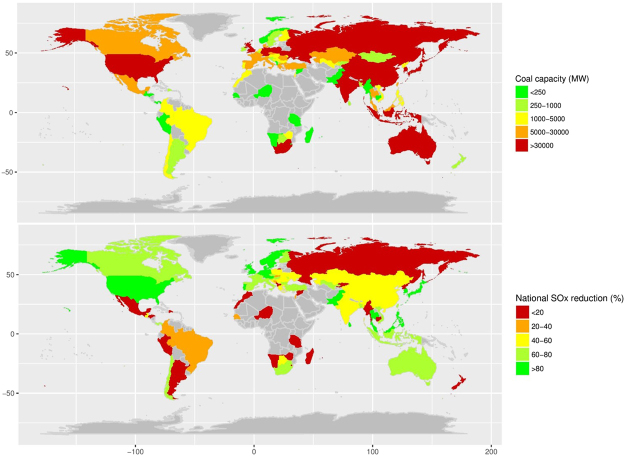
Total coal capacity (upper panel) and national SO_x_ reduction by country (lower panel) in 2012. The map is created by using R version 3.2.5, Package ‘rworldmap’^30^.

To calculate SO_x_ emission controls, the efficiencies of different SO_x_ reduction control systems in coal-fired power plants were extracted from the literature. Most SO_x_ control systems in the studied countries had relatively high SO_x_ reduction efficiency, by 80% or more (Supplementary Table 1). Data on SOx control technology were only available for larger power plants. As a consequence, 19 countries had no data on control technologies. However, the total capacity of plants with missing control technology data is only 14.15 GW, representing 0.78% of the total coal capacity in the study. We assigned those missing as 0 reduction in the following analysis. We defined national SO_x_ reduction as the average SO_x_ reduction percentage weighted by generating capacities of individual plants in a given country. Total coal capacities and national SO_x_ reduction in included countries in 2012 are summarized in Fig. 1. The lack of installing control systems in small units in many countries produced a bimodal distribution of national SO_x_ reduction, with a median of 58.49% (Supplementary Figure 1).

To determine effects of SO_x_ emission controls on CVD, we extracted age- and sex-adjusted CVD incidence rates from the Global Burden of Disease (GBD) Study^22^, including two subcategories: ischemic heart diseases (IHD) and rheumatic heart diseases (RHD). The former reflects coronary artery disease, which may have a stronger association with air pollution; the latter is a contagious disease that we used as a falsifying outcome. Table 2 summarizes these and other covariates included in the study. IHD was more common among males, while RHD, accounting for less than 1% of CVD, was more common among females. One behavior risk factor for CVD—smoking prevalence—was almost three times higher among males than females.

**Table 2:** Mean, range, and 95% CI of covariates among studied countries in 2012.

	**Mean**	**Range**	**95% CI**
**CVD incidence** ^a^			
Males	873.90	(293.16~1994.66)	(780.30~967.51)
Females	820.61	(305.85~1819.81)	(731.31~909.91)
**Ischemic heart diseases**			
Males	461.28	(156.69~859.76)	(422.06~500.50)
Females	311.61	(89.37~603.3)	(285.75~337.48)
**Rheumatic heart diseases**			
Males	5.42	(0.63~32.32)	(4.10~6.75)
Females	7.04	(0.72~42.60)	(5.07~9.02)
SO_x_ reduction^b^	47.00	(0.00~95.00)	(39.50~54.51)
**Smoking prevalence** ^b^			
Males	29.61	(8.80~57.00)	(27.24~31.99)
Females	11.80	(0.70~34.70)	(9.74~13.86)
Per capita GDP (PPP)^c^	20.44	(0.84~63.8)	(17.01~23.88)
Traffic index^b^	61.29	(17.99~97.73)	(56.76~65.82)
Industrialization^b^	18.67	(2.55~62.09)	(16.45~20.90)
Coal capacity^d^	22726.01	(10.10~780959.5)	(1495.29~43956.73)
**Population** ^e^			
All	72150.04	(623.86~1355386.95)	(25955.69~118344.39)
Males	36378.31	(308.06~697964.30)	(12550.04~60206.57)
Females	35771.73	(315.80~657422.65)	(13401.46~58142.00)

^a^Unit: incident case per 100,000.

^b^Unit: %.

^c^Unit: thousands United States Dollars.

^d^Unit: megawatt.

^e^Unit: thousand people.

We applied a Poisson regression to analyze the relative risk of age-standardized CVD incidence associated with national SO_x_ reduction, adjusted for behavior, economic, and regional factors. A 10% decrease in SO_x_ emission from coal-fired power plants is associated with a 0.75% lower standardized CVD incidence rate [relative risk (RR) = 0.9925, 95% confidence interval (CI) = 0.9892–0.9959], after adjustments (primary model, sex combined, Table 3). The association of SO_x_ reduction was stronger for lower CVD in females (RR = 0.9831, 95% CI = 0.9762–0.9901) than in males (RR = 0.9972, 95% CI = 0.9905–1.0039).

**Table 3:** The effects of sulfate oxide controls in coal-fired power plants on cardiovascular diseases in different models, among sex combined, males, and females.

	**Primary model**		**Univariate model**		**Behavior model**		**Economics model**		**Non-regional model**	
**Sex combined**	RR	95%CI	RR	95%CI	RR	95%CI	RR	RR	95%CI	95%CI
Intercept	0.0041	(0.0038~0.0043)	0.0063	(0.0002~0.1931)	0.0060	(0.0059~0.0061)	0.0049	(0.0047~0.0052)	0.0049	(0.0046~0.0051)
SO_x_ reduction ^a^	0.9925	(0.9892~0.9959)	1.0529	(0.6090~1.8205)	0.9847	(0.9815~0.9878)	0.9861	(0.9824~0.9898)	0.9793	(0.9756~0.9830)
Smoking prevalence ^b^	1.0037	(1.0033~1.0041)			1.0070	(1.0066~1.0075)			1.0068	(1.0063~1.0072)
Per capita GDP(PPP) ^c^					1.0092	(1.0087~1.0097)	1.0083	(1.0071~1.0095)	1.0095	(1.0083~1.0107)
Traffic index ^b^	1.0018	(1.0011~1.0024)					1.0017	(1.0011~1.0024)	1.0005	(0.9998~1.0011)
Industrialization ^b^	1.0038	(1.0026~1.005)					1.0032	(1.0018~1.0047)	1.0022	(1.0008~1.0037)
Ln coal capacity ^d^	1.0450	(1.0413~1.0487)					1.0177	(1.0146~1.0208)	1.0159	(1.0128~1.0190)
Region										
Africa	0.7909	(0.7389~0.8464)								
America	0.8082	(0.7655~0.8533)								
Europe	1.5187	(1.4466~1.5945)								
South-East Asia	0.8046	(0.7693~0.8415)								
Western Pacific	0.8099	(0.7715~0.8502)								
Eastern Mediterranean	1.0000	—								
**Males**										
Intercept	0.0043	(0.0038~0.0049)	0.0065	(0.0001~0.7692)	0.0052	(0.0050~0.0055)	0.0049	(0.0045~0.0054)	0.0053	(0.0048~0.0058)
SO_x_ reduction ^a^	0.9972	(0.9905~1.0039)	1.0546	(0.4910~2.2652)	0.9775	(0.9710~0.9840)	0.9898	(0.9825~0.9972)	0.9686	(0.9612~0.9761)
Smoking prevalence ^b^	1.0032	(1.0016~1.0048)			1.0104	(1.0092~1.0116)			1.0104	(1.0090~1.0117)
Per capita GDP(PPP) ^c^					1.0117	(1.0106~1.0128)	1.0085	(1.0061~1.0109)	1.0148	(1.0123~1.0173)
Traffic index ^b^	1.0012	(0.9999~1.0026)					1.0011	(0.9998~1.0023)	0.9976	(0.9963~0.9990)
Industrialization ^b^	1.0019	(0.9994~1.0043)					1.0046	(1.0017~1.0075)	1.0004	(0.9975~1.0033)
Ln coal capacity ^d^	1.0489	(1.0417~1.0562)					1.0203	(1.0144~1.0264)	1.0108	(1.0048~1.0169)
Region										
Africa	0.7261	(0.6342~0.8314)								
America	0.8101	(0.7272~0.9024)								
Europe	1.4162	(1.2879~1.5573)								
South-East Asia	0.7813	(0.7164~0.8521)								
Western Pacific	0.7753	(0.7040~0.8539)								
Eastern Mediterranean	1.0000	—								
**Females**										
Intercept	0.0039	(0.0034~0.0044)	0.0062	(0.0001~0.8218)	0.0058	(0.0056~0.0060)	0.0049	(0.0044~0.0055)	0.0027	(0.0024~0.0030)
SO_x_ reduction ^a^	0.9831	(0.9762~0.9901)	1.0512	(0.4797~2.3036)	0.9983	(0.9919~1.0049)	0.9821	(0.9746~0.9895)	0.9898	(0.9822~0.9973)
Smoking prevalence ^b^	1.0124	(1.0079~1.0168)			1.0347	(1.0320~1.0375)			1.0485	(1.0452~1.0517)
Per capita GDP(PPP) ^c^					0.9965	(0.9950~0.9981)	1.0080	(1.0056~1.0105)	0.9951	(0.9926~0.9976)
Traffic index ^b^	1.0010	(0.9996~1.0025)					1.0025	(1.0011~1.0038)	1.0000	(0.9986~1.0013)
Industrialization ^b^	1.0072	(1.0047~1.0097)					1.0017	(0.9987~1.0047)	1.0113	(1.0082~1.0143)
Ln coal capacity ^d^	1.0417	(1.0343~1.0492)					1.0149	(1.0086~1.0212)	1.0461	(1.0392~1.0531)
Region										
Africa	0.8605	(0.7504~0.9867)								
America	0.7909	(0.707~0.8847)								
Europe	1.5105	(1.3538~1.6854)								
South-East Asia	0.8229	(0.7493~0.9038)								
Western Pacific	0.8689	(0.7853~0.9614)								
Eastern Mediterranean	1.0000	—								

^a^Unit: 10%.

^b^Unit: %.

^c^Unit: thousands United States Dollars.

^d^Unit: Natural log of MW.

Results of analysis of IHD and RHD as different outcomes are presented in Table 4. Unlike the effects on CVD, the effects of SO_x_ reduction on IHD were stronger in males than in females. A 10% decrease in SO_x_ emission from coal-fired power plants was associated with 0.9722-fold (95% CI = 0.9643–0.9801) lower IHD incidence among males, while females had an analogous association of 0.9882 (95% CI = 0.9783–0.9981). No statistically significant relationships between SO_x_ reduction and RHD incidence rate among either males or females were found.

**Table 4:** The effects of sulfur oxide controls in coal-fired power plants on the incidence of ischemic heart disease and rheumatic heart disease, among sex combined, males, and females, in the primary model.

	Ischemic heart disease		Rheumatic heart disease	
Sex combined	RR	95%CI	RR	95%CI
Intercept	0.0107	(0.0096~0.0120)	0.0006	(0.0004~0.0010)
SO_x_ reduction^a^	0.9739	(0.9679~0.9800)	0.9691	(0.9408~0.9984)
Smoking prevalence^b^	1.0099	(1.0091~1.0107)	0.9917	(0.9884~0.9950)
Traffic index^b^	0.9847	(0.9833~0.9860)	0.9673	(0.9607~0.9739)
Industrialization^b^	0.9987	(0.9964~1.0010)	1.0170	(1.0066~1.0274)
Ln coal capacity^c^	1.0318	(1.0253~1.0382)	1.1302	(1.0981~1.1633)
Region				
Africa	0.6639	(0.5980~0.7370)	0.3802	(0.2475~0.5841)
America	0.6673	(0.6079~0.7326)	0.1734	(0.1084~0.2773)
Europe	0.8163	(0.7533~0.8846)	0.1628	(0.1088~0.2437)
South-East Asia	0.5692	(0.5308~0.6104)	0.2765	(0.2094~0.3652)
Western Pacific	0.4523	(0.4190~0.4882)	0.1938	(0.1430~0.2626)
Eastern Mediterranean	1.0000	—	1.0000	—
Males				
Intercept	0.0114	(0.0098~0.0133)	0.0004	(0.0001~0.0014)
SO_x_ reduction^a^	0.9722	(0.9643~0.9801)	0.9738	(0.9108~1.0410)
Smoking prevalence^b^	1.0067	(1.0046~1.0089)	1.0052	(0.9849~1.0259)
Traffic index^b^	0.9857	(0.9839~0.9876)	0.9629	(0.9446~0.9816)
Industrialization^b^	0.9972	(0.9941~1.0004)	1.0151	(0.9918~1.0390)
Ln coal capacity^c^	1.0388	(1.0299~1.0477)	1.1540	(1.0717~1.2426)
Region				
Africa	0.6654	(0.5796~0.7641)	0.3902	(0.1517~1.0038)
America	0.6884	(0.6065~0.7814)	0.2347	(0.0789~0.6980)
Europe	0.8850	(0.7962~0.9837)	0.1670	(0.0703~0.3969)
South-East Asia	0.5698	(0.5184~0.6262)	0.2349	(0.1163~0.4747)
Western Pacific	0.4312	(0.3868~0.4808)	0.1556	(0.0662~0.3660)
Eastern Mediterranean	1.0000	—	1.0000	—
Females				
Intercept	0.0099	(0.0083~0.0117)	0.0008	(0.0003~0.0019)
SO_x_ reduction^a^	0.9882	(0.9783~0.9981)	0.9500	(0.8969~1.0064)
Smoking prevalence^b^	0.9827	(0.9758~0.9896)	1.0208	(0.9650~1.0798)
Traffic index^b^	0.9886	(0.9864~0.9909)	0.9628	(0.9498~0.9760)
Industrialization^b^	0.9990	(0.9954~1.0026)	1.0180	(0.9989~1.0374)
Ln coal capacity^c^	1.0178	(1.0080~1.0277)	1.1351	(1.0764~1.1971)
Region				
Africa	0.6625	(0.5654~0.7764)	0.3681	(0.1655~0.8187)
America	0.6212	(0.5397~0.7151)	0.1511	(0.0593~0.3855)
Europe	0.9703	(0.8480~1.1104)	0.1102	(0.0403~0.3009)
South-East Asia	0.6014	(0.5409~0.6688)	0.2629	(0.1558~0.4438)
Western Pacific	0.4908	(0.4365~0.5519)	0.1870	(0.1051~0.3326)
Eastern Mediterranean	1.0000	—	1.0000	—

^a^Unit: 10%.

^b^Unit: %.

^c^Unit: Natural log of MW.

**Figure 2:**
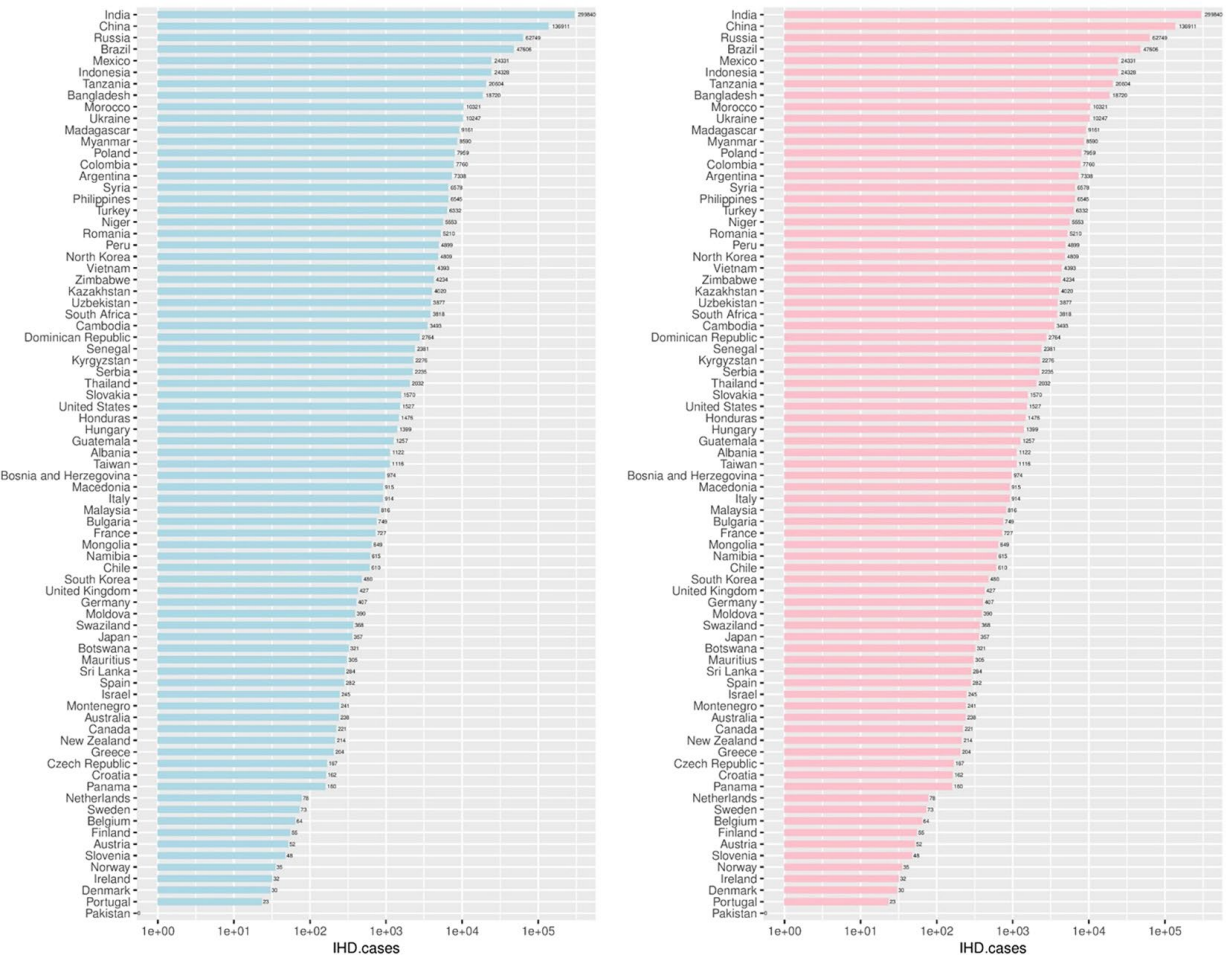
The IHD incidence cases attributable to suboptimal SO_x_ emission control in studied countries among males (left panel) and females (right panel) in 2012.

CVD incident cases attributable to suboptimal emission controls were estimated in all studied countries, assuming every country can reach 95% emission reduction. The fractions of CVD attributable to suboptimal SO_x_ reduction (PAF) were up to 1.43% and 8.00% for males and females, respectively (Supplementary Table 2). Similarly, the PAFs of IHD from suboptimal SO_x_ reduction were up to 13.24% and 5.70% for males and females, respectively. The number of attributable cases varied widely between countries. Take IHD for example, India and China had the highest preventable cases from optimizing SO_x_ reductions in coal-fired power plants, with estimations of 381,843 and 177,756 preventable cases, respectively (Fig. 2).

## Discussion

To our knowledge, this is the first study showing the preventable CVD incidence attributable to SO_x_ reductions from coal-fired power plants from a global perspective. We found that 10% reductions in SO_x_ emissions were associated with CVD incidence rates that were 0.28% lower for males and 1.69% lower for females. Up to 13.24% and 5.70% of incident IHD cases are attributable to suboptimal SO_x_ emissions control in coal-fired power plants among males and females, respectively, given a country can reach 95% SO_x_ reduction in the electricity sector. Our falsifying test (see Methods) revealed no relationship between RHD and air pollution, supporting SO_x_ as a risk factor on air pollution related CVD.

Taking SO_x_ reduction in coal-fired power plants as a determinant of CVD incidence was reasonable and adequate from several perspectives: (1) The majority of SO_2_ emission was from fossil fuel combustion, mostly coal-fired power units. Therefore, using the reduction percentage in coal-fired plants could capture the largest amount of SO_x_ reduction. (2) The implication of national SO_x_ reduction provides an alternative for policy application at the national level. By summarizing a national SO_x_ reduction, policy makers could use the results presented here to help estimate the counterfactual outcome given a country has improved its SO_x_ control system in coal-fired power plants. (3) Our approach provides a direct method to estimate the externality costs from coal-fired power plants, specifically from SO_x_ control systems, by comparing the costs of treatment for CVD attributable to SO_x_ emissions from coal-fired power plants.

Considering the magnitude of estimated costs of CVD, retrofitting FGD equipment in coal-fired power plants could be economically justifiable. Take the US as an example: the national SO_x_ reduction is 82.60% in the US. The US needs to install FGD in a total capacity of 42,093.37 MW (=339462.7 MW*(95%–82.6%)) to reach optimal reduction. Given the the cost of installing FGD at $455 per kW and 30 years lifetime of coal-fired power plants^19^, the annual cost of SO_x_ emissions control would be $638.42 million, nominal price. In contrast, the estimated cost of CVD is $1,067.96 million (=564.32 billion dollars*(2,756/1,456,342))^2,23^ under the estimated PAF = 0.0003 for males and 0.002 for females, respectively, in the US (Supplementary Table 2). Yet, for many countries, the situation is more nuanced. For example, China has much higher CVD incidence and PAF than the US, so the health benefits per unit of SO_x_ reduction could be much higher, making FGD installation a cost-effective strategy to improve public health. Moreover, developing countries usually have relatively low SO_x_ reduction rates, such as in the cases of China (59.44%) and India (44.45%). Marginal costs of FGD might rise, while marginal benefits might decrease, when these countries increase their SO_x_ reduction rates. It is possible to find an efficient level of SO_x_ reduction rates (below 95%) when the marginal costs equal marginal benefits. The above examples illustrate the applications of SO_x_ reduction rate and PAF as helpful analytical tools to illuminate policy-making in public health and SOx emissions control.

The log-linear model also provides an interpretation of elasticity. For example, the elasticity of IHD on demanding SO_x_ emission control systems is 0.07 (=ln(0.9722) × 2.5) and 0.03 (=ln(0.9882) × 2.5) among males and females, respectively, given the national SO_x_ reduction is 25% in a given country *ceteris paribus* (Table 4). This implies the change of IHD is more sensitive to SO_x_ reduction among males than females. Similarly, the elasticity is 0.21 (=ln(0.9722) × 7.5) and 0.09 (=ln(0.9882) × 7.5) among males and females, respectively, given the national SO_x_ reduction is 75% in a given country *ceteris paribus*. The elasticity becomes larger when SO_x_ reduction improves, which means the incidence of IHD would be even more sensitive to additional improvement of the emission controls for countries having already had better SO_x_ control systems in coal-fired power plants.

Several limitations or concerns should also be addressed. (1) The cross-sectional study did not provide a temporal interpretation of the causal effect of SO_x_ reduction on CVD prevention. However, since the national SO_x_ reduction in 2012 remained relatively constant compared to our 2016 data, it could be deemed as a marker for what has happened over many previous years. (2) Despite using an ecological study design, the potential for “ecological fallacy”^24^ is unlikely because our analysis on aggregated data is meant to inform policy decisions at the national level and for international comparison, but not at the individual level^25^. (3) This approach can be regarded as conservative in the sense that some of these plants may have actually reduced emissions more than our approach recognizes, implying that our approach actually underestimates the association between SO_x_ and CVD/IHD. Countries with national SO_x_ controls equal to 0 had lower CVD incidence rates (631 vs. 960 cases per hundred thousand males, on average). However, note that plants with missing control data amount to less than 1% of the total global coal capacity, so different assumptions about these missing data are not expected to have a meaningful impact on the analysis. (4) The study did not adjust for meteorological, geographical and/or other covariates^26^. If we assume the lack of considering meteorological effect misclassified our exposure, we might underestimate the true effect as well. Other covariates, such as socioeconomic status has an impact on cardiovascular disease at individual^27^ and national levels^28^. Also, we’ve adjusted per capita GDP(PPP) and the geographic region as proxy indicators of healthcare expenditure and living standard. However, similar to previous report^29^, we didn’t find any statistically significant relationship between coal capacity and socioeconomic status at the national level. (5) It is noteworthy that even though the study does not explicitly calculate the effects of detailed secondary formation and/or byproduct of SO2, retrofitting SOx control system reduces both SO2 and its secondary products. It is the cumulative effect that is of interest in this study. We did not consider the influence of seasonal differences on SOx emission, either. Instead of the ambient SOx levels, we focused on the reduction (control) effect of SOx emission (%) from coal-fired power plants, which is assumably constant across seasons. (6) The outcome data were obtained and interpolated from the GBD estimation^22^. Although we acknowledged the possible inconsistency of over- or under-reports from the global incidence data, GBD estimation provided the most thorough CVD incidences that we could access for a better international comparison.

## Conclusion

In conclusion, CVD is a common, costly, and often fatal condition. Improvement in SO_x_ controls in coal-fired power plants has a marked association with lower incidence of CVD and IHD. Although the causality and biological mechanisms need further exploration, SO_x_ emission is a pervasive public health issue with major cardiovascular and healthcare economic consequences. Since SO_x_ emission is primarily from coal combustion, regulations on SO_x_ emission do present a key target for national and international intervention.

## Methods

### Data source

A total of 79 countries with data available for analyses were included in 2012. We obtained the age- and sex-adjusted CVD incidence rates from the Global Burden of Disease (GBD) Study^22^. CVD data included two subcategories, ischemic heart diseases (IHD) and rheumatic heart diseases (RHD). The former was coded as 410–410.9, 411–411.1, 411.8–411.9 in the International Classification of Diseases version 9 (ICD-9) and I21.0–I21.4, I21.9, I22.0–I22.2, and I22.8–I22.9 in ICD-10. The latter was coded as 391.0–391.2, 391.8–391.9, 392.0, 394.0–394.2, 394.9, 395.0–395.2, 395.9, 396.0–396.3, 396.8–396.9, 397.0–397.1, 397.9, 398.8–398.9 in ICD-9 and I01.0–I01.2, I01.8–I01.9, I02.0, I05.0–I05.2, I05.8–I05.9, I06.0–I06.2, I06.8–I06.9, I07.0–I07.2, I07.8–I07.9, I08.0–I08.3, I08.8–I08.9, I09.0–I09.2, I09.8–I09.9 in ICD-10^22^. The GBD study has thorough estimation of incidence rates of CVD in 2010 and 2015, respectively. For CVD incidence rates data between 2011 and 2014, we estimated using linear interpolation.

Coal capacity is defined as the generating capacity of a coal-fired power plant [unit: megawatt (MW)]. The estimation for reduction of each unit is based on the representative SO_x_ reduction percentage of the corresponding control technology from literature review, summarized in Supplementary Table 1. National SO_x_ reduction is the coal-capacity-weighted average SO_x_ reduction in a given country. The formula is written as follows:

$$National\,S{O}_{{\rm{x}}}\,reduction\,( \% )=\frac{{\sum }_{i=1}^{n}\,S{O}_{{\rm{x}}}\,reductio{n}_{i}( \% )\times coal\,capacit{y}_{i}\,(MW)}{{\sum }_{i=1}^{n}\,coal\,capacit{y}_{i}(MW)}$$

where *i* is a coal-fired power unit and *n* is the total units in a country. Coal capacities are the weight for the reductions in different power units. Missing data on SO_x_ control are assigned 0. Data on the coal capacity of every plant were derived from the Utility Data Institute World Electric Power Plants Data Base (UDI WEPP)^21^.

We also collected data exclusively on both behavior and economic covariates at the national level, including smoking prevalence, economy, traffic index, and macroeconomic indicators, and industrialization. Annual smoking prevalence within each country was estimated and sex- and age-adjusted^31^. The macro level indicator was the annual per capita gross domestic product adjusted for purchasing power parity [GDP (PPP)] and inflation to the base year 2011 to capture a country’s standard of living level^32^. Traffic index, measured as the proportion of a country’s population living in urban areas, was applied to capture air pollutants emitted from all mechanical vehicles and public transports associated with human activities^33^. The industrialization level was measured using the shares of CO_2_ emissions from manufacturing industries and construction in total CO_2_ emissions (% of total fuel combustion)^34^. We further grouped studied countries into 6 WHO regions (combination of geographical distribution and mortality): Africa, the Americas, Southeast Asia, Europe, Eastern Mediterranean, and the Western Pacific^35^.

### Data analysis

We took a natural logarithm of coal capacity to approximate normal distribution in the model. A Poisson regression was performed for count data of incidence cases of diseases. Our primary model is as following:

$$\begin{array}{rcl}ln(E[{\lambda }_{i}]) & = & {\beta }_{0}+{\beta }_{1}\times S{O}_{2}\,reductio{n}_{i}+{\beta }_{2}\times Smoking\,prevalenc{e}_{i}\\  &  & +{\beta }_{3}\times Traffic\,inde{x}_{i}+{\beta }_{4}\times Industrializatio{n}_{i}+{\beta }_{5}\\  &  & \times log{(Nationalcoalcapacity)}_{i}+{\beta }_{6}\times I\_Regio{n}_{i}\end{array}$$

where *i* denotes each country; *ln*(*E*[*λ*_*i*_]) denotes the natural log of expected standardized incident rates for CVD conditioned on covariates *X*_*i*_; ${\beta }_{0}$ is the intercept; ${\beta }_{1}to\,{\beta }_{6}$ are coefficients of individual covariates; and *I_Region* is indicator variable for the six WHO regions to consider the underlying difference in hygiene and healthcare status.

In addition to the above primary model, four other models were specified to assess sensitivity to the inclusion of different adjustment covariates. They were: (1) Univariate model with SO_x_ reduction only; (2) Behavior-adjusted model with smoking prevalence and healthcare index of per capita GDP (PPP); (3) Economic-adjusted model with per capita GDP (PPP), traffic index, and industrialization; and (4) non-regional model with combination of behavior and economics. All models were analyzed for both sexes combined, males, and females, respectively, and weighted by nationwide sex-specific population^33^ for all multivariate models.

Under the assumption that every country could hypothetically reach an optimal national SO_x_ reduction by a factor of 95%, we estimated the proportional attributable factor (PAF) for IHD for every country. The formula for PAF is written as follows^36^:

$$PAF=\frac{{P}_{i}\times (R{R}_{i}-1)}{1+{P}_{i}\times (R{R}_{i}-1)}$$

where *P*_*i*_ is the proportion of people exposed to suboptimal SO_x_ reduction. In the estimation, we applied WHO mortality strata and assumed the *P*_*i*_ is 0.1 in strata A countries and 0.5 in stratum B to E countries^37^, respectively, as often used in other studies^38–40^. $R{R}_{i}$ is the relative risks from the primary model, comparing existing national SO_x_ reduction in 2012 vs. the counterfactual optimal reduction (95%). Supplementary Table 2 shows the step-by-step calculation for PAFs. The incident cases of IHD attributable to SO_x_ controls in coal-fired power plants were estimated by multiplying the standardized incidence rates by sex-specific population and PAF.

$$SOx\,associated\,IHD=Population\times {\rm{Standardized}}\,{\rm{Incidence}}\,{\rm{Rate}}\times PAF$$

We performed the PROC GENMOD procedure with a log link function, using SAS version 9·4 (SAS Institute, Cary, NC, US) to estimate the effect of selected factors on standardized incidences of CVD, IHD, and RHD, respectively.

### Additional analysis and falsification test

To investigate the possibility that general health improvements correlated with SO_x_ reduction in coal-fired power plants might be obscuring our CVD results, we further analyzed two subcategories of CVD: IHD and RHD. Since the latter is related to previously unsatisfactorily-treated streptococcus infection, we identified RHD as a falsification outcome that might be a marker that are not expected to bear any relationship to air pollution. We applied the primary model for IHDs and rheumatic heart diseases, respectively, as the additional analysis and examined whether the relationship between RHD and SO_x_ reduction existed as a falsification test.

## Electronic supplementary material

Supplementary tables and figure
