# Supplementary material for: A Global Perspective on Sulfur Oxide Controls in Coal-Fired Power Plants and Cardiovascular Disease
Source: Sci Rep. 2018 Feb 8;8:2611. doi: 10.1038/s41598-018-20404-2 (PMC5805744; doi:10.1038/s41598-018-20404-2)
Supplement: Supplementary file 1 — Supplementary tables and figure [file 41598_2018_20404_MOESM1_ESM.pdf]

TITLE:

A Global Perspective on Sulfur Oxide Controls in Coal-Fired Power Plants and Cardiovascular Disease

AUTHORS:

Cheng-Kuan Lin[1], Ro-Ting Lin[2], Pi-Cheng Chen[3], Pu Wang[4], Nathalie De Marcellis-Warin [5], Corwin Zigler[6], David C. Christiani[1,7]

1 Department of Environmental Health, Harvard T.H. Chan School of Public Health, 665 Huntington Avenue, Building 1, Room 1406, Boston, Massachusetts 02115, USA
2 Department of Occupational Safety and Health, China Medical University, 91 Hsueh-Shih Road, Taichung 40402, Taiwan
3 Department of Environmental Engineering, Cheng Kung University, 1 University Road, Tainan City 701, Taiwan
4 Belfer Center for Science and International Affairs, John F. Kennedy School of Government, Harvard University, 79 JFK Street, Cambridge, MA 02138, USA
5 Department of Mathematics and Industrial Engineering, Polytechnique Montréal, 2900, boul. Édouard-Montpetit, Montréal (Québec) H3T 1J4, Canada
6 Department of Biostatistics, Harvard T.H. Chan School of Public Health, 655 Huntington Avenue, Building 2, 4th Floor, Boston MA 02115, USA
7 Department of Epidemiology, Harvard T.H. Chan School of Public Health, 665 Huntington Avenue, Building 1, Room 1401, Boston, Massachusetts 02115, USA

Supplementary Figure 1. Distribution of national reduction in sulfur oxide (SO_X) emissions

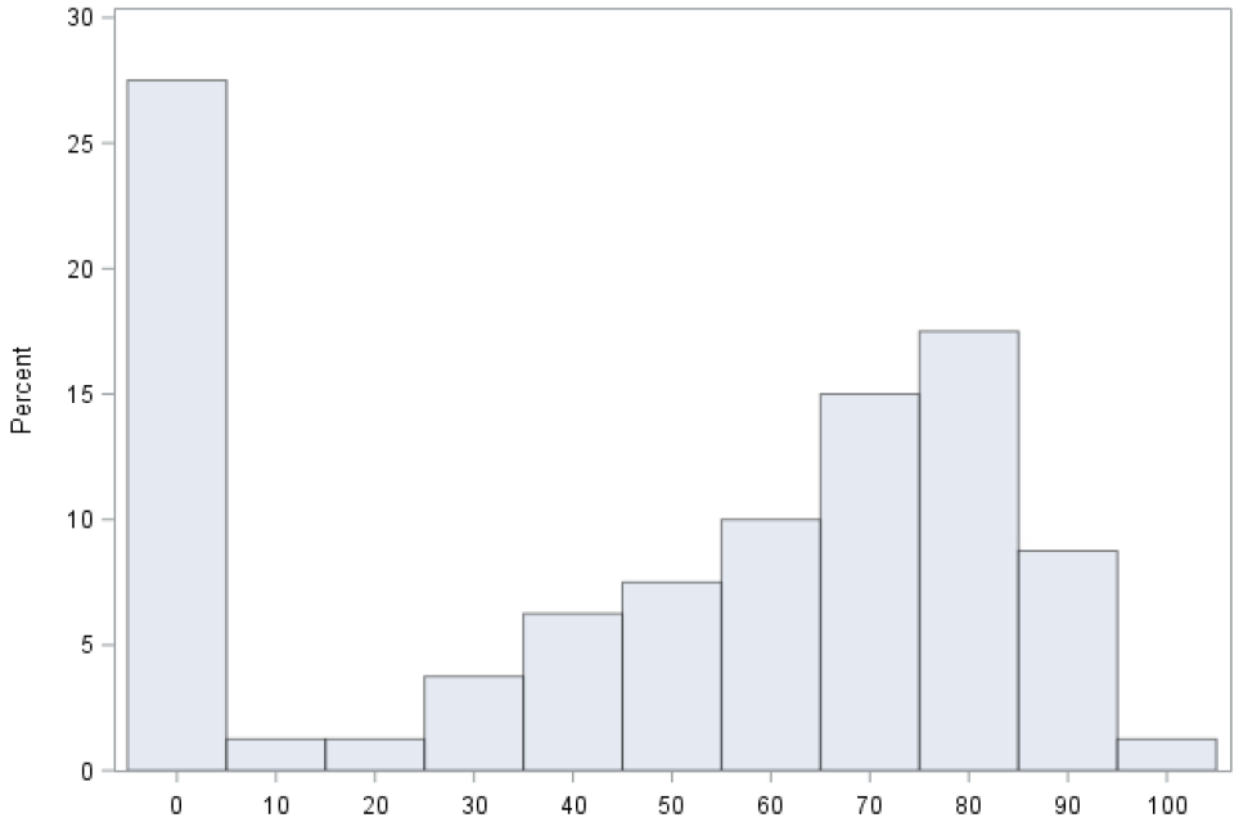

Legend: X-axis unit: percentage

Supplementary table 1 Reduction percentage of sulfur oxide control systems in coal-fired power plants

Reduction percentage	Emission control system
>= 95%	Noxso Corp or NOXSO process, Regenerative activated coke technology system (SO_2 and NO_X control), Flash dryer absorber system, Wellman-Lord process for FGD, sodium-sulfite based, Atmospheric circulating fluidized bed boiler, also used to code for SO2CTL for ACFB units, Compliance fuel/bubbling fluidized bed boiler, Combined SO_X and NO_X removal system, Pressurized fluidized-bed combustor, Spray-dry scrubber with activated carbon injection, Spray dry FGD with activated carbon injection, SNOX flue gas cleaning system, Wet limestone FGD plus activated carbon injection for mercury control
90%~95%	CANSOLV (regenerable aqueous amine FGD system), Circulating-bed FGD scrubber, aka Circoclean, Circulating dry FGD scrubber, First generation wet sulfuric acid FGD system developed by Chiyoda Corp, Wet limestone bubbling reactor FGD system developed by Chiyoda Corp, licensed elsewhere, Double alkali FGD scrubber, Dry FGD with activated carbon injection, Wet/dry lime spray FGD system, FGD scrubber (unspecified), Wet limestone FGD scrubber design, Lime injection, Limestone injection, Magnesium oxide FGD scrubber, Novel integrated desulphurization scrubber, generally supplied by Alstom (dry lime), NID FGD scrubber with activated carbon injection, Reflux circulating fluidized bed FGD scrubber with activated carbon injection, Spray dry FGD scrubber (typically using lime reagent), Spray dry FGD scrubber system, Spray dry circulating FGD, Spray dry rotary-atomizer FGD, Semi-dry lime FGD or other semidry gas cleaning system, Dry sorbent injection (typically lime or limestone) with activated carbon, Dry sorbent injection (typically lime or limestone) for acid gas or mercury control, Simplified Wet FGD (FGD design), Seawater FGD scrubber, Trona injection system for SO_2 control, Wet calcium carbonate FGD scrubber, Wet carbide sludge FGD scrubber, Wet FGD (unspecified), Wet FGD with sorbent injection for mercury control, Wet lime FGD scrubber, Wet lime-alkaline fly ash FGD scrubber, Wet lime/limestone FGD scrubber, Wet lime/magnesium FGD scrubber, Wet limestone FGD scrubber, Wet limestone FGD with sorbent injection for mercury control, Wet soda ash FGD scrubber, Wet sodium carbonate FGD scrubber, Wet scrubber (unspecified)
80%-90%	Ammonia or ammonium sulfate FGD scrubber, Bubbling fluidized bed boiler, Coal blending, Semi-dry circulating fluidized-bed FGD scrubber, Turbosorp scrubber, Semi-dry circulating fluidized-bed FGD scrubber/activated carbon injection, Dry aqueous carbonate FGD scrubber, Dry FGD scrubber (unspecified), Dry lime FGD scrubber, hydrated lime injection, Dry lime FGD scrubber, Hydrated lime injection with activated carbon or carbon filters, Dry scrubber, Reflux circulating fluidized bed FGD scrubber (semi-dry design)
<80%	Compliance fuel (fuel or fuels that allow plant to meet applicable air quality standards), Compliance fuel for SO_2 control, activated carbon injection for mercury control, Dalkia/Clarke, Limestone injection into furnace with CAO activation, Dry scrubber with additional sorbent injection for mercury control, Coal washing

Supplementary table 2. Estimated CVD and IHD incident cases attributable to suboptimal SO_2 controls in coal-fired power plants

				CVD				IHD			
Country	Strata	Sex	Population	Incidence	RR	PAF	Cases	Incidence	RR	PAF	Cases
Albania	B	Female	1436.63	948.0166	1.175434	0.080643	1098.32	332.6994	1.120829	0.056972	272.309
Argentina	B	Female	21503.12	512.0833	1.175434	0.080643	8879.937	138.2494	1.120829	0.056972	1693.673
Australia	A	Female	11453.16	596.1879	1.05574	0.005543	378.4986	135.7733	1.039021	0.003887	60.44336
Austria	A	Female	4322.8	1146.167	1.01978	0.001974	97.8099	251.4756	1.013919	0.00139	15.10961
Bangladesh	D	Female	76841.37	523.5315	1.055616	0.027056	10884.15	451.6289	1.038935	0.019096	6626.898
Belgium	A	Female	5644.75	1003.844	1.018798	0.001876	106.3196	235.4248	1.01323	0.001321	17.55785
Bosnia and Herzegovina	B	Female	1922.5	1349.059	1.10076	0.047964	1243.971	378.5058	1.070096	0.033861	246.4012
Botswana	E	Female	1067.29	414.3959	1.086453	0.041435	183.2599	294.6526	1.060262	0.029249	91.98368
Brazil	B	Female	102776.8	653.5399	1.123265	0.058054	38994.45	292.071	1.085489	0.040993	12305.18
Bulgaria	B	Female	3750.7	1819.806	1.034603	0.017007	1160.825	422.5572	1.024297	0.012003	190.2268
Cambodia	B	Female	7601.34	434.9973	1.175434	0.080643	2666.518	285.1735	1.120829	0.056972	1234.993
Canada	A	Female	17573.55	506.5908	1.039706	0.003955	352.0876	112.2493	1.02786	0.002778	54.80387
Chile	B	Female	8812.06	449.0074	1.044512	0.021771	861.4258	94.74023	1.03121	0.015365	128.2794
China	B	Female	657422.7	700.6195	1.062369	0.030242	139293.8	291.0682	1.043621	0.021345	40844.56
Colombia	B	Female	23779.63	537.3459	1.114032	0.053941	6892.487	322.2299	1.079185	0.038085	2918.254
Croatia	A	Female	2220.1	1641.567	1.067848	0.006739	245.6016	408.4573	1.047416	0.004719	42.7948
Czech Republic	A	Female	5365.17	1562.365	1.029876	0.002979	249.6866	446.0002	1.020992	0.002095	50.12648
Denmark	A	Female	2821.74	948.4362	1.016656	0.001663	44.50046	240.9256	1.011725	0.001171	7.961936
Dominican Republic	B	Female	5089.68	546.4754	1.175434	0.080643	2242.998	339.5946	1.120829	0.056972	984.728

Finland	A	Female	2759.47	1132.373	1.032421	0.003232	100.9806	242.2363	1.022772	0.002272	15.18731
France	A	Female	32612.88	864.7902	1.051859	0.005159	1455.044	156.0029	1.036324	0.003619	184.1362
Germany	A	Female	41004.3	1257.307	1.014896	0.001487	766.8374	269.0345	1.01049	0.001048	115.5947
Greece	A	Female	5671.2	1134.841	1.062683	0.006229	400.9089	226.2468	1.043838	0.004365	56.00309
Guatemala	D	Female	7857.16	305.8522	1.094385	0.045066	1082.985	220.9685	1.065719	0.031814	552.3477
Honduras	B	Female	3869.12	419.2517	1.175434	0.080643	1308.142	353.301	1.120829	0.056972	778.7932
Hungary	C	Female	5224.77	1646.099	1.050101	0.024438	2101.815	420.8019	1.035101	0.017248	379.2129
India	D	Female	608395.9	580.7915	1.089801	0.042971	151838.6	444.3354	1.062566	0.030334	82003.18
Indonesia	B	Female	123023.8	585.1935	1.046935	0.022929	16507.51	395.3609	1.032898	0.016183	7871.139
Ireland	A	Female	2341.03	598.7613	1.02585	0.002578	36.14122	187.2539	1.018174	0.001814	7.952419
Israel	A	Female	3888.91	552.2559	1.103438	0.010238	219.8759	170.4386	1.071932	0.007142	47.33768
Italy	A	Female	30725	1137.151	1.052391	0.005212	1820.952	215.937	1.036694	0.003656	242.5618
Japan	A	Female	65248.03	789.7363	1.021489	0.002144	1104.952	89.37061	1.015118	0.001509	88.02237
Kazakhstan	C	Female	8704.79	997.3705	1.087462	0.041899	3637.603	548.851	1.060957	0.029577	1413.073
Kyrgyzstan	B	Female	2859.59	712.2386	1.175434	0.080643	1642.469	506.4876	1.120829	0.056972	825.159
Macedonia	E	Female	1038.72	1160.361	1.175434	0.080643	971.9844	373.8786	1.120829	0.056972	221.2555
Madagascar	D	Female	11185.48	476.2166	1.175434	0.080643	4295.631	452.8014	1.120829	0.056972	2885.542
Malaysia	B	Female	14643.78	452.7075	1.016588	0.008226	545.3192	348.5941	1.011678	0.005805	296.3342
Mauritius	D	Female	635.31	689.6669	1.175434	0.080643	353.3401	260.5811	1.120829	0.056972	94.31778
Mexico	B	Female	61356.42	436.2411	1.175434	0.080643	21585.12	249.4816	1.120829	0.056972	8720.947
Moldova	C	Female	2114.75	1085.014	1.039555	0.019394	445.0032	353.1582	1.027755	0.013687	102.2229

Mongolia	B	Female	1416.31	676.6552	1.08641	0.041416	396.9071	552.5462	1.060232	0.029236	228.7907
Montenegro	E	Female	315.8	1232.065	1.175434	0.080643	313.7714	325.4218	1.120829	0.056972	58.54958
Morocco	D	Female	16729.86	595.8472	1.175434	0.080643	8038.87	378.9147	1.120829	0.056972	3611.593
Myanmar	D	Female	26888.9	495.2436	1.175434	0.080643	10738.9	185.8823	1.120829	0.056972	2847.581
Namibia	E	Female	1179.11	417.027	1.175434	0.080643	396.5394	272.2096	1.120829	0.056972	182.8617
Netherlands	A	Female	8448.54	882.9135	1.016605	0.001658	123.6596	206.078	1.01169	0.001168	20.32932
New Zealand	A	Female	2261.65	692.0615	1.172589	0.016966	265.5531	183.0352	1.118914	0.011752	48.6472
Niger	D	Female	8752.39	318.5734	1.175434	0.080643	2248.558	296.7433	1.120829	0.056972	1479.696
North Korea	D	Female	12661.38	757.933	1.175434	0.080643	7738.908	266.6374	1.120829	0.056972	1923.389
Norway	A	Female	2501.19	866.7973	1.021024	0.002098	45.48486	213.2068	1.014791	0.001477	7.876089
Panama	D	Female	1864.78	495.4316	1.039555	0.019394	179.1761	245.4675	1.027755	0.013687	62.65292
Peru	B	Female	15091.89	409.406	1.175434	0.080643	4982.71	190.0625	1.120829	0.056972	1634.199
Philippines	D	Female	47460.39	521.6102	1.025829	0.01275	3156.284	452.8583	1.018159	0.008998	1933.872
Poland	B	Female	19929.08	1533.488	1.065471	0.031698	9687.2	471.7375	1.04577	0.022373	2103.367
Portugal	B	Female	5507.9	1148.982	1.008544	0.000854	54.02133	265.6694	1.006022	0.000602	8.806075
Romania	A	Female	10269.04	1675.739	1.087629	0.041976	7223.256	443.641	1.061072	0.029631	1349.926
Russia	B	Female	76810.58	1673.768	1.143144	0.066792	85869.31	493.7753	1.099011	0.04717	17890.41
Senegal	C	Female	7027.06	358.2085	1.101841	0.048453	1219.643	306.0559	1.070838	0.034207	735.6847
Serbia	D	Female	4594.36	1583.284	1.08439	0.040487	2945.071	450.0703	1.058841	0.02858	590.9629
Slovakia	E	Female	2790.32	1202.691	1.109546	0.051929	1742.673	339.2174	1.076117	0.036663	347.0242
Slovenia	B	Female	1039.39	1361.105	1.045342	0.004514	63.85665	348.9872	1.031789	0.003169	11.49429

South Africa	A	Female	26900.78	660.7661	1.04024	0.019723	3505.83	353.3818	1.028232	0.01392	1323.242
South Korea	E	Female	24946.71	491.4082	1.015178	0.007532	923.3148	107.5745	1.010687	0.005315	142.641
Spain	B	Female	23641.93	894.8819	1.026422	0.002635	557.5256	174.296	1.018574	0.001854	76.39721
Sri Lanka	A	Female	10530.37	653.7116	1.008544	0.004254	292.8098	308.987	1.006022	0.003002	97.67073
Swaziland	B	Female	624.76	446.7208	1.175434	0.080643	225.0698	352.3253	1.120829	0.056972	125.4071
Sweden	E	Female	4783.81	1105.629	1.02004	0.002	105.7803	248.541	1.014101	0.001408	16.74185
Syria	A	Female	9877.31	387.4803	1.175434	0.080643	3086.428	354.5751	1.120829	0.056972	1995.317
Taiwan	B	Female	11642.5	667.9627	1.039454	0.019346	1504.457	244.2822	1.027684	0.013653	388.3024
Tanzania	B	Female	24484.47	445.9267	1.175434	0.080643	8804.85	483.4825	1.120829	0.056972	6744.294
Thailand	E	Female	34016.01	708.4309	1.022367	0.01106	2665.142	263.3032	1.015733	0.007805	699.0552
Turkey	B	Female	38068.52	614.7887	1.046147	0.022553	5278.409	336.825	1.03235	0.015917	2040.995
Ukraine	B	Female	24367.26	1798.855	1.078803	0.037908	16616.25	484.9034	1.054988	0.026758	3161.697
United Kingdom	C	Female	32296.93	916.5584	1.020999	0.002096	620.3132	237.5749	1.014774	0.001475	113.1913
United States	A	Female	158948.6	682.1426	1.021319	0.002127	2306.571	178.3509	1.014998	0.001498	424.534
Uzbekistan	A	Female	14528.69	628.6064	1.06019	0.029216	2668.222	467.7144	1.04211	0.020621	1401.231
Vietnam	B	Female	45692.84	649.6496	1.035247	0.017318	5140.752	280.4182	1.024747	0.012222	1566.026
Zimbabwe	E	Female	7378.07	356.9898	1.162583	0.07518	1980.168	335.7487	1.112167	0.053105	1315.514
Albania	B	Male	1444.04	1212.813	1.028914	0.014251	249.5881	586.6516	1.305222	0.132405	1121.663
Argentina	B	Male	20592.11	555.4942	1.028914	0.014251	1630.163	269.1305	1.305222	0.132405	7337.814
Australia	A	Male	11458.21	721.3166	1.009611	0.00096	79.36159	224.278	1.093506	0.009264	238.0691
Austria	A	Male	4132.68	1147.374	1.00346	0.000346	16.40108	383.7217	1.032805	0.00327	51.85251

Bangladesh	D	Male	78416.02	594.7236	1.00959	0.004772	2225.602	535.6515	1.093294	0.044568	18720.2
Belgium	A	Male	5434.77	1048.155	1.00329	0.000329	18.73324	381.1493	1.031167	0.003107	64.36098
Bosnia and Herzegovina	B	Male	1905.92	1516.399	1.017074	0.008465	244.6371	647.4209	1.171408	0.078939	974.051
Botswana	E	Male	1065.53	437.8371	1.01473	0.007311	34.10805	442.0412	1.146422	0.068217	321.3069
Brazil	B	Male	99624.81	755.1746	1.02071	0.010249	7710.669	500.4346	1.211136	0.095488	47606.07
Bulgaria	B	Male	3553.04	1994.66	1.006017	0.002999	212.5724	752.2874	1.057661	0.028022	749.0136
Cambodia	B	Male	7230.92	413.7959	1.028914	0.014251	426.4129	364.8571	1.305222	0.132405	3493.168
Canada	A	Male	17294.6	651.7526	1.00689	0.000689	77.61155	193.9813	1.066272	0.006584	220.867
Chile	B	Male	8576.38	532.684	1.007709	0.00384	175.4265	198.2241	1.074407	0.035869	609.7868
China	B	Male	697964.3	819.8808	1.010726	0.005335	30527.07	393.8039	1.104845	0.049811	136911.3
Colombia	B	Male	23101.39	529.5706	1.019226	0.009521	1164.812	378.5253	1.194775	0.088745	7760.257
Croatia	A	Male	2066.92	1717.613	1.011644	0.001163	41.28878	694.5043	1.11425	0.011296	162.1513
Czech Republic	A	Male	5179.99	1552.708	1.005205	0.00052	41.84131	650.1549	1.04971	0.004946	166.5845
Denmark	A	Male	2779.24	1063.527	1.002917	0.000292	8.620235	393.1528	1.027596	0.002752	30.07003
Dominican Republic	B	Male	5065.36	595.2152	1.028914	0.014251	429.6699	412.1656	1.305222	0.132405	2764.3
Finland	A	Male	2665.18	1155.499	1.005643	0.000564	17.36693	386.0834	1.053988	0.00537	55.25441
France	A	Male	30948.92	891.618	1.008956	0.000895	246.91	272.6403	1.086889	0.008614	726.8461
Germany	A	Male	39473.65	1298.837	1.002611	0.000261	133.8281	418.9371	1.024667	0.002461	406.9125
Greece	A	Male	5438.46	1179.098	1.010779	0.001077	69.04572	359.3702	1.105382	0.010428	203.8132
Guatemala	D	Male	7511.6	293.1578	1.016032	0.007952	175.1187	225.6057	1.160248	0.07418	1257.106
Honduras	B	Male	3867.01	380.0417	1.028914	0.014251	209.4388	288.2117	1.305222	0.132405	1475.672

Hungary	C	Male	4733.57	1655.07	1.008658	0.00431	337.6979	734.1844	1.083897	0.04026	1399.153
India	D	Male	655193.7	688.6374	1.015281	0.007582	34210.8	646.9255	1.15225	0.07074	299840
Indonesia	B	Male	125014.1	638.0974	1.008121	0.004044	3226.123	515.1483	1.078517	0.037776	24327.76
Ireland	A	Male	2326.84	722.6209	1.004511	0.000451	7.581175	319.6768	1.042956	0.004277	31.81552
Israel	A	Male	3805.6	692.5598	1.017509	0.001748	46.0673	372.6577	1.176107	0.017306	245.4307
Italy	A	Male	29012.72	1169.195	1.009046	0.000904	306.57	362.1395	1.087795	0.008703	914.4083
Japan	A	Male	61891.8	868.3823	1.003757	0.000376	201.8202	162.5391	1.03566	0.003553	357.4582
Kazakhstan	C	Male	8116.67	917.8064	1.014896	0.007393	550.7367	718.0521	1.148178	0.068978	4020.193
Kyrgyzstan	B	Male	2788.64	691.5559	1.028914	0.014251	274.8339	616.5091	1.305222	0.132405	2276.328
Macedonia	E	Male	1030.55	1366.235	1.028914	0.014251	200.6527	670.7576	1.305222	0.132405	915.2456
Madagascar	D	Male	11108.24	503.3256	1.028914	0.014251	796.7918	622.8854	1.305222	0.132405	9161.285
Malaysia	B	Male	14378.16	560.2147	1.002905	0.001451	116.8464	418.772	1.027483	0.013555	816.1922
Mauritius	D	Male	623.02	752.6469	1.028914	0.014251	66.82576	369.7969	1.305222	0.132405	305.0481
Mexico	B	Male	60714.54	450.5739	1.028914	0.014251	3898.606	302.6634	1.305222	0.132405	24330.76
Moldova	C	Male	1960	1156.163	1.006864	0.003421	77.51126	622.2282	1.066017	0.031954	389.6992
Mongolia	B	Male	1392.03	716.5069	1.014723	0.007308	72.88547	683.5988	1.146348	0.068185	648.8369
Montenegro	E	Male	308.06	1381.742	1.028914	0.014251	60.66146	590.5636	1.305222	0.132405	240.8824
Morocco	D	Male	16254.34	631.9166	1.028914	0.014251	1463.793	479.5488	1.305222	0.132405	10320.6
Myanmar	D	Male	25654.94	530.9697	1.028914	0.014251	1941.294	252.8733	1.305222	0.132405	8589.681
Namibia	E	Male	1112.54	426.1546	1.028914	0.014251	67.56682	417.5239	1.305222	0.132405	615.0352
Netherlands	A	Male	8300.78	993.3111	1.002908	0.000291	23.9742	341.3324	1.027512	0.002744	77.73664

New Zealand	A	Male	2174.24	901.6272	1.028475	0.002839	55.66217	337.2858	1.30002	0.029128	213.6079
Niger	D	Male	8883.39	394.0691	1.028914	0.014251	498.8864	472.0997	1.305222	0.132405	5552.844
North Korea	D	Male	12101.98	597.3016	1.028914	0.014251	1030.151	300.1489	1.305222	0.132405	4809.458
Norway	A	Male	2517.18	1047.397	1.003676	0.000367	9.687679	402.0075	1.034882	0.003476	35.17549
Panama	D	Male	1878.98	512.1484	1.006864	0.003421	32.91604	266.1825	1.066017	0.031954	159.8179
Peru	B	Male	15066.88	423.1158	1.028914	0.014251	908.5172	245.5731	1.305222	0.132405	4898.996
Philippines	D	Male	48556.93	589.0865	1.004507	0.002248	643.1612	641.5885	1.04292	0.021009	6545.075
Poland	B	Male	18680.41	1633.741	1.011246	0.005592	1706.512	816.1393	1.110166	0.052207	7959.4
Portugal	B	Male	5007.12	1061.784	1.001501	0.00015	7.980705	328.3564	1.014118	0.00141	23.17957
Romania	A	Male	9675.92	1821.362	1.014924	0.007406	1305.271	779.1578	1.148469	0.069105	5209.853
Russia	B	Male	66476.96	1581.859	1.023873	0.011796	12403.82	859.7572	1.246661	0.10979	62749.46
Senegal	C	Male	6753.05	366.3644	1.01725	0.008551	211.5602	442.105	1.173304	0.079742	2380.751
Serbia	D	Male	4388.22	1753.068	1.01439	0.007143	549.5375	763.9817	1.142838	0.066658	2234.729
Slovakia	E	Male	2625.18	1373.119	1.0185	0.009165	330.3861	699.8922	1.186856	0.085445	1569.918
Slovenia	B	Male	1023.5	1485.079	1.007851	0.000784	11.92339	618.6222	1.075814	0.007524	47.64119
South Africa	A	Male	25936.5	583.3448	1.006981	0.003479	526.3051	453.0044	1.067175	0.032496	3818.07
South Korea	E	Male	24661.75	494.1631	1.00266	0.001328	161.8693	156.685	1.025135	0.012412	479.6007
Spain	B	Male	22995.16	874.8691	1.00461	0.000461	92.6901	280.2877	1.043914	0.004372	281.7985
Sri Lanka	A	Male	9891.49	713.5176	1.001501	0.00075	52.94107	409.7195	1.014118	0.00701	284.0848
Swaziland	B	Male	606.94	420.0358	1.028914	0.014251	36.33144	457.9608	1.305222	0.132405	368.0248
Sweden	E	Male	4759.65	1346.091	1.003505	0.00035	22.4492	462.085	1.033239	0.003313	72.86172

Syria	A	Male	10101.45	449.0501	1.028914	0.014251	646.4413	491.8048	1.305222	0.132405	6577.783
Taiwan	B	Male	11673.32	731.0352	1.006847	0.003412	291.1618	299.8653	1.065847	0.031874	1115.725
Tanzania	B	Male	24161.24	483.8605	1.028914	0.014251	1666.057	644.0696	1.305222	0.132405	20604.16
Thailand	E	Male	33148.12	770.8165	1.003908	0.00195	498.3502	336.4214	1.037126	0.018225	2032.357
Turkey	B	Male	36780.67	632.743	1.007988	0.003978	925.7582	463.3225	1.07718	0.037156	6331.932
Ukraine	B	Male	20952.69	1648.774	1.013466	0.006688	2310.473	783.4735	1.133151	0.06242	10246.74
United Kingdom	C	Male	31276.84	1017.431	1.003672	0.000367	116.7919	393.3242	1.034841	0.003472	427.1197
United States	A	Male	155850.9	774.1997	1.003727	0.000373	449.5184	277.9723	1.035374	0.003525	1527.099
Uzbekistan	A	Male	14063.76	621.3741	1.01036	0.005154	450.3594	572.9078	1.101112	0.048123	3877.389
Vietnam	B	Male	44642.71	569.426	1.006127	0.003054	776.4211	344.8591	1.058746	0.028535	4393.044
Zimbabwe	E	Male	7187.41	389.5593	1.026922	0.013282	371.8881	477.036	1.28179	0.123495	4234.217

CVD: cardiovascular diseases; IHD: ischemic heart diseases; PAF=population attributable factor; RR: relative risk.
